# Development of a novel *in vitro* strategy to understand the impact of shaving on skin health: combining tape strip exfoliation and human skin equivalent technology

**DOI:** 10.3389/fmed.2023.1236790

**Published:** 2023-11-02

**Authors:** Lydia Costello, Kirsty Goncalves, Victoria Maltman, Nicole Barrett, Kous Shah, Alison Stephens, Tereasa Dicolandrea, Ilaria Ambrogio, Erica Hodgson, Stefan Przyborski

**Affiliations:** ^1^Department of Biosciences, Durham University, Durham, United Kingdom; ^2^Procter & Gamble, Reading, Berkshire, United Kingdom; ^3^Procter & Gamble, Cincinnati, OH, United States; ^4^Reprocell Europe Ltd., Glasgow, United Kingdom

**Keywords:** exfoliation, *stratum corneum*, skin equivalent, shaving, inflammation, tape strip

## Abstract

**Introduction:**

The removal of unwanted hair is a widespread grooming practice adopted by both males and females. Although many depilatory techniques are now available, shaving remains the most common, despite its propensity to irritate skin. Current techniques to investigate the impact of shaving regimes on skin health rely on costly and lengthy clinical trials, which hinge on recruitment of human volunteers and can require invasive biopsies to elucidate cellular and molecular-level changes.

**Methods:**

Well-characterised human skin equivalent technology was combined with a commonplace dermatological technique of tape stripping, to remove cellular material from the uppermost layer of the skin (*stratum corneum*). This method of exfoliation recapitulated aspects of razor-based shaving *in vitro*, offering a robust and standardised *in vitro* method to study inflammatory processes such as those invoked by grooming practices.

**Results:**

Tape strip insult induced inflammatory changes in the skin equivalent such as: increased epidermal proliferation, epidermal thickening, increased cytokine production and impaired barrier function. These changes paralleled effects seen with a single dry razor pass, correlated with the number of tape strips removed, and were attenuated by pre-application of shaving foam, or post-application of moisturisation.

**Discussion:**

Tape strip removal is a common dermatological technique, in this study we demonstrate a novel application of tape stripping, to mimic barrier damage and inflammation associated with a dry shave. We validate this method, comparing it to razor-based shaving *in vitro* and demonstrate the propensity of suitable shave- and skin-care formulations to mitigate damage. This provides a novel methodology to examine grooming associated damage and a platform for screening potential skin care formulations.

## 1. Introduction

The acceptance of body hair declined throughout the 20th century, and hair removal remains a common beauty practice amongst both men and women. Facial shaving is the most common cosmetic practice in men, and 70% of men are reported to use a wet-shaving technique with a frequency of four to five times per week (1). Body hair removal has also been reported in over 99% of women, with shaving of the axilla and legs being most prevalent (2, 3). However, the act of shaving, and therefore consumer satisfaction, is a continuous balance between achieving the desired “smoothness” from removal of hairs, while minimizing the negative impact to the underlying skin due to nicks, skin dryness, and inflammation which can lead to skin sensitivities (4, 5).

Effective shaving is technically challenging, as it requires the robust removal of stiff terminal hairs, whilst minimizing collateral damage to the underlying skin substrate (6). Poor-quality shaving can cause nicks, cuts and a significant inflammatory response, mediated by the high concentration of immune cells, blood vessels and nerve endings within the perifollicular skin (7). In the face of barrier damage, pro-inflammatory cytokine release and mast cells degranulation (8), leads to hyperproliferation of epidermal keratinocytes, and stimulation of free nerve endings supports development of itch and erythema (9). This has both acute and chronic impacts on skin health, ranging from barrier dysfunction (10, 11), razor burn (12), pseudofolliculitis (13), epidermal thickening (14), and turnover of supporting cell types such as Merkel cells (15). Shaving-related irritation is the most common male cosmetic consumer complaint reported by 88% of men, which is associated with erythema and unpleasant sensations of burning, stinging or itching (Gillette, unpublished).

To ameliorate the unwanted side effects of shaving and ensure consumer satisfaction whilst achieving the desired smooth/hairless end point, a plethora of skin care regimes have been designed to guarantee a pleasant consumer experience, with 90% of men reporting the frequent use of a basic skin care regimen (16). Pre-shave preparation is often recommended prior to commencing the act of shaving, which involves cleansing, exfoliation and hydration to improve the act of shaving and consumer experience (17). A combination of razor design (e.g., number of blades, presence of lubricating strip, etc.) and use of lubricating gels/foams (6), aim to reduce friction and minimize biomechanical forces that damage skin during the act of shaving (5).

However, despite the use of pre- and in-shave products to minimize damage, epidermal barrier dysfunction (10) and decreased hydration (18) have still been reported post-shave. Post-shave products mainly aim at restoring moisturization and improving skin hydration. Use of moisturizing formulations is commonplace among male shavers with 37.8% of men reporting the use of post-shave moisturization in their grooming routine. Moisturizing formulations including emollients and high levels of glycerine have been reported to repair barrier function 1 h after shaving compared with non-moisturizing controls (17). Therefore, despite the inflammatory reactions that can occur through shaving, modern fashion dictates consumer habit and drives the need for skincare formulations that repair barrier damage and negate unwanted side effects induced through shaving.

Current approaches to better understand the cellular and molecular basis for shave-induced irritation rely on time consuming and costly clinical trials (8, 18–20) that often hinge on the recruitment of willing participants, which may be a limiting factor in terms of sample size, hindering statistical power and robustness of results. This is particularly a problem due to the incidence of potential irritation, resulting in a negative perception amongst consumers. For these reasons, research in this area is shifting towards *in vitro* methodologies that avoid the use of human participants. However, these are not without their limitations. Currently, *ex vivo* porcine skin models are often used to investigate the impact of shaving on skin (11). Porcine skin contains hair follicles and characterization has demonstrated it reacts to shaving insult in a similar manner to human skin, however species-specific differences limit its value when applying knowledge gained through these studies to consumer scenarios.

In addition to hair removal, corneocytes account for approximately 20% of the material removed through shaving in the male facial area (21), but this is as high as 36% for the female axillary region (14) Therefore we hypothesize that other mechanical methods of exfoliation that remove material from the *stratum corneum* may recapitulate aspects of inflammatory response that arises due to shaving. Tape strip removal is a common dermatological technique, whereby adhesive tapes are applied to the surface of the skin and subsequently removed (22, 23). Removal of the tapes results in the adhesion of lipidic and cellular material from the *stratum corneum* to the tape, which has many dermatological applications, such as: induction of barrier dysfunction (24–27), downstream analysis of *stratum corneum* composition (28, 29), structural assessment of *stratum corneum* organization (28) and even drug distribution (30). This hypothesis is supported by a study by Dabboue et al. (11), who characterized tape strip insult in *ex vivo* porcine skin against razor damage and found that removal of 30 tape strips equated to barrier dysfunction in the region of 3 dry razor passes. Although there are many variables to consider, this study provides proof of concept that other mechanical methods of exfoliation or corneocyte removal may be able to recapitulate aspects of shaving induced damage *in vitro*.

Previously we have described the development and characterization of a fully humanized skin equivalent (HSE), bioengineered *in vitro* to study skin health applications (31–33). This system is advantageous as it utilizes endogenous extracellular matrix (ECM) secreted from dermal fibroblasts within a 3D culture environment, providing a solid dermal foundation upon which a stratified and organized epidermis is constructed. This also eliminates the use of any animal-derived exogenous ECM products, ensuring a fully humanized system. We have reported many applications and adaptations of this HSE to investigate skin health such as: ultraviolent damage (34), skin pigmentation (34), neurosensory irritation (35), and screening of actives (34, 36).

In this study, we have developed an *in vitro* approach to mimic shaving-induced irritation and inflammation through combination of our well established HSE with a tape stripping methodology. We have characterized the correlation between number of tape strips removed with cellular and molecular inflammation responses to investigate the impact of different intensities of mechanical damage on skin health. We also examine the link between tape strip removal and shaving, to establish an *in vitro* approach that mimics diffuse shaving-induced barrier damage and recapitulate the damaging aspects this has on skin health. Furthermore, we also demonstrate the ability of this platform to screen potential skincare regimes or interventions that mitigate and attenuate barrier damage in these circumstances. Therefore, the novel approach described in this study offers a unique combination of technologies to advance our understanding of skin barrier damage, particularly in relation to shaving and offers strategies to overcome such damage.

## 2. Materials and methods

### 2.1. Generation of skin equivalents

Commercially available cells were used to create HSEs including human neonatal keratinocytes #1817888, #1944927, #2288858 and #2018512 (HEKn, Thermo Fisher Scientific), and neonatal dermal fibroblasts #1366356 and #1366434 (HDFn, Thermo Fisher Scientific).

HSEs were generated as previously described (32). Briefly, HDFn were seeded onto Alvetex^®^ Scaffold (Reprocell Europe Ltd., Glasgow, United Kingdom) at a density of 0.5 × 10^6^ cells per scaffold and incubated with DMEM, 2 mM L-glutamine and 10% FBS for 14 days. HEKn were then seeded at a density of 1.3 × 10^6^ onto each dermal compartment and cultured for 2 days in submerged culture to promote proliferation in Epilife^®^ Medium (Thermo Fisher Scientific) supplemented with Human Keratinocyte Growth Supplement (HKGS, Thermo Fisher Scientific), 10 ng mL^−1^ Keratinocyte Growth Factor (KGF, PeproTech, London, United Kingdom), 140 μM CaCl_2_ (Sigma-Aldrich) and 10 mg mL^−1^ Ascorbic Acid (Sigma-Aldrich). HSEs were then raised to the air-liquid interface (ALI) and maintained for a further 14 days in high calcium conditions to promote keratinocyte differentiation and epidermal stratification.

### 2.2. Tape stripping

Tape stripping of HSEs has previously been described (34). D-Squame^®^ tape strips (CuDerm, Texas, United States) were applied to the surface of HSEs with even pressure by use of the D500-D-squame pressure instrument that ensures a standardised pressure of 225 gcm^−2^. Tape strips were removed using forceps.

### 2.3. Razor pass

HSEs were unclipped from their cell culture inserts, placed in a sterile Petri dish and held with forceps while a Gillette Men’s Mach 3 disposable razor was passed once across the surface. This was done in the absence of water or lubricant. HSEs were then returned to their plastic-ware and further cultured for 4 days prior to analysis.

### 2.4. Topical moisturising cream application

Topical application of a glycerin-rich basal moisturising formulation (35, 36) was administered following tape strip insult. Four microliters of formulation was added using a positive displacement pipette to the surface of HSEs following tape strip removal and spread evenly across the surface using a glass rod. After 4 days samples were harvested for analysis.

### 2.5. Topical shaving foam application

Prior to razor passing on selected models, a pre-application of Gillette classic sensitive shaving foam was administered. Ten microliters of formulation was added using a positive displacement pipette to the surface of HSEs and spread evenly across the surface using a glass rod. After 4 days samples were harvested for analysis.

### 2.6. Transepidermal water loss measurement

Transepidermal water loss (TEWL) was measured using the VapoMeter (Delfin Technologies). HSEs were equilibrated at room temperature for 20 min prior to measurement.

### 2.7. Cytokine array

Samples of culture medium were harvested from HSEs 4 days following treatment. Analysis of the cytokine content of the medium was performed by Eve Technologies (Calgary, Canada) using the Human Cytokine Proinflammatory Focused 15-Plex Discovery Assay^®^ Array (HDF15).

### 2.8. Paraffin wax embedding

Samples were fixed in 4% paraformaldehyde (Sigma-Aldrich) overnight at 4°C, then dehydrated through a series of ethanols. Samples were incubated in Histoclear (Scientific Laboratory Supplies, Nottingham, United Kingdom) alone, then in a mix of 50:50 histoclear:paraffin wax (Thermo Fisher Scientific) followed by paraffin wax alone. Samples were embedded in paraffin wax in plastic moulds (CellPath, Newton, United Kingdom) and sectioned transversely using a microtome (Leica RM2125RT). Five micrometers sections were captured onto charged microscope slides (Thermo Fisher Scientific).

### 2.9. Haemotoxylin & eosin staining

Samples were deparaffinised in Histoclear (Scientific Laboratory Supplies) and rehydrated through a series of ethanol baths. Samples were then incubated in Mayer’s haematoxylin (Sigma-Aldrich) for 5 min before being treated with alkaline ethanol for 30 s to blue the nuclei. Slides were dehydrated through a series of ethanols, incubated with eosin (Sigma-Aldrich) for 30 s and further dehydrated in ethanol. Finally, slides were cleared in Histoclear and mounted with coverslips using Omnimount mountant (Scientific Laboratory Supplies).

### 2.10. Immunofluorescence

Sections were deparaffinised in Histoclear and rehydrated through a series of ethanols. Antigen retrieval was performed in citrate buffer pH 6 (Sigma-Aldrich) at 95°C for 20 min, followed by blocking and permeabilization for 1 h with a solution containing: 20% neonatal calf serum (NCS, Sigma-Aldrich) and 0.4% Triton X-100 (Sigma-Aldrich) in phosphate buffered saline (PBS). Samples were then incubated overnight at 4°C in primary antibody diluted in blocking buffer (ki67, Abcam, Cambridge, United Kingdom, ab16667, 1:100, Filaggrin, Abcam, ab17808, 1:100). Slides were washed three times in PBS and incubated with the appropriate secondary antibody diluted in blocking buffer for 1 h at room temperature (donkey anti-rabbit Alexa^®^ Fluor 488, Thermo Fisher Scientific, 1:1000) and washed three times in PBS. Finally, slides were mounted using Vectashield Hardset with DAPI (Vector Laboratories, Peterborough, United Kingdom).

### 2.11. Microscopy

Histology images were captured using Leica ICC50 high-definition camera and Brightfield microscope. Immunofluorescence images were taken using the Zeiss 880 confocal microscope with Airyscan and Zen software.

### 2.12. Micro-BCA protein analysis

The amount of material removed from the surface of the models following a razor pass was measured using a Micro-BCA protein analysis kit (Thermo Fisher) following the manufacturers guidelines. In brief, the material was removed from the razor blades via washing in PBS. Collected samples were centrifuged to pellet material and then lysed in 5% SDS. Lysates were incubated with the working BCA solution at 37°C for 2 h before being read on the plate reader at 600 nm alongside protein standards.

### 2.13. Biometric measurements

Epidermal thickness and ki67 counts were achieved using Image J software^1^ and a previously described methodology (37). The thickness of the viable epidermis was measured from the basement membrane to the top of the *stratum granulosum* from H&E stained images, using the straight line tool function. ki67-positive cells were identified on immunofluorescence images and labelled using the multi-point tool.

### 2.14. Statistical analysis

GraphPad Prism software was used to measure the statistical significance using a one-way ANOVA or student’s *t*-test as appropriate. * = *p* ≤ 0.05, ** = *p* ≤ 0.01, *** = *p* ≤ 0.001, and **** = *p* ≤ 0.0001.

## 3. Results

### 3.1. Removal of tape strips reduces skin barrier function *in vitro*

**Figure 1:**
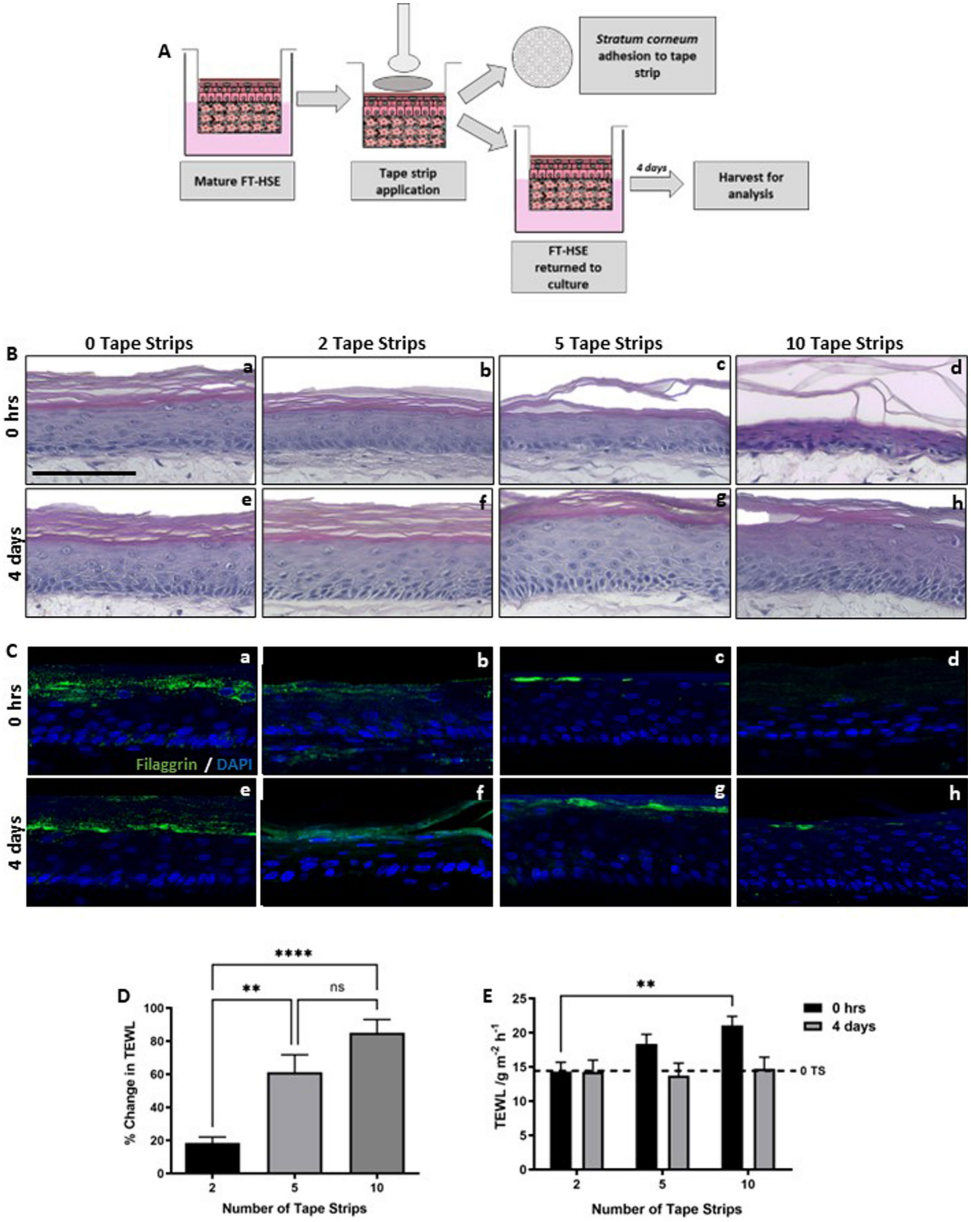
Number of tape strips removed correlates with level of barrier damage. Schematic protocol depicting the adhesion and removal of tape strips from FT-HSEs *in vitro* **(A)**. Tape strips were applied with controlled pressure using a spring-loaded applicator and removed from the surface of HSEs using forceps. Cellular material is visible on tape strips upon removal and models were either analysed immediately or returned to culture for 4 days prior to analysis. H&E staining **(B)** of HSEs immediately following insult **(Ba–c)** ranging from 0–10 tape strip removal, or following a 4 days recovery period **(Be–g)**. Immunofluorescence of terminal differentiation marker Filaggrin **(C)** stained in green and nuclei are highlighted by DAPI in blue. Demonstrates changes in barrier biomarker expression immediately following insult **(Ca–d)** and following 4 days recovery **(Ce–h)**. Transepidermal water loss (TEWL) increased with increasing number of tape strips immediately following insult **(D)** (data represent mean ± SEM, *n* = 9, one-way ANOVA with Tukey’s multiple comparison test). TEWL recovered over a 4 days period **(E)** (data represent ± SEM, *n* = 9, two-way ANOVA with Tukey’s multiple comparison test) to a level comparable to 0 tape strip controls (dashed line). Scale bar: 100 μm. ** = *p* < 0.01 and **** = *p* < 0.0001.

Tape strip removal is a common dermatological technique, used routinely in dermatology clinics to harvest skin surface components such as lipids for analysis or to damage the barrier function (23, 38–40). Here, we outline its use to induce barrier dysfunction *in vitro* using a fully humanized and well-characterized bioengineered HSE. We utilize the technique of tape stripping to induce diffuse barrier damage to the surface of HSEs and track subsequent protein-level changes up to 4 days post-insult (Figure 1A).

A number of tape strips were removed sequentially from HSEs (0, 2, 5 10 tape strips), before being returned to culture, to mimic mild/moderate/extreme barrier disruption. HSEs displayed good viability following tape strip removal, with no visible damage observed through histological analysis immediately following insult (Figures 1Ba–c), even following the most severe condition of 10 sequential tape strips. Epidermal morphology appeared as expected in all conditions, well-stratified and structured epidermises consisting of a *stratum basale*, suprabasal viable layers and visible *stratum corneum*. Even after the most severe insult (10 tape strips), the epidermis appeared viable and adhered to the dermal compartment via the basement membrane. Following a 4 days recovery period epidermal thickening was observed (Figures 1Be–g), particularly following 5 tape strip removal. Keratinocyte hyperproliferation and epidermal thickening is a known response to mechanical trauma (41), and a consequence of the inflammatory cascade known to arise post-tape strip removal (42).

Immunofluorescence analysis of filaggrin (Figures 1Ca–h), a terminal keratinocyte differentiation marker that is indicative of an intact skin barrier, revealed a reduction in positive staining with increasing number of tape strips immediately following insult. Filaggrin expression then appeared to re-establish 4 days following insult, however in the most extreme condition of 10 tape strip removal, expression was patchy and inconsistent across the length of the epidermis.

Transepidermal water loss (TEWL) is a well characterized measure of skin barrier function, often used in dermatology clinics and clinical trials, an increase in TEWL correlates with impaired barrier function. TEWL increased immediately following tape strip removal (Figure 1D) and correlated with number of tape strips removed. TEWL then decreased following a further 4 days in culture (Figure 1E) and returned to baseline levels (dashed line) after even the most severe insult of 10 tape strips. This suggests that HSEs withstand significant insult robustly and recover quickly from barrier damage.

### 3.2. Tape strip insult induces keratinocyte proliferation through induction of a pro-inflammatory response

**Figure 2:**
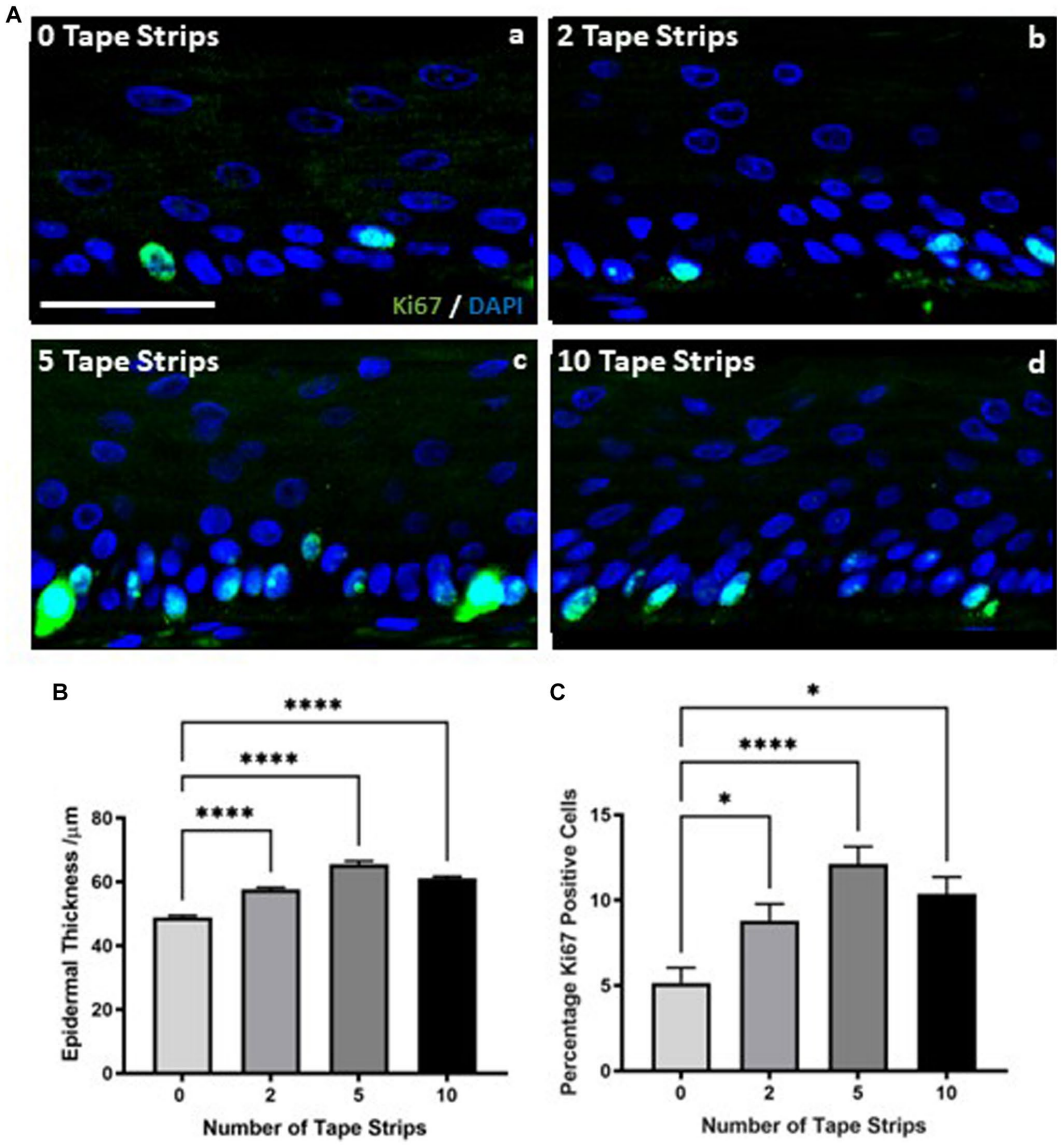
Increased keratinocyte proliferation drives epidermal thickening following tape strip insult. Representative immunofluorescence images **(A)** of FT-HSEs 4 days after removal of 0–10 tape strips. ki67 is stained in green and DAPI highlights nuclei in blue. Epidermal thickness **(B)** (data represent mean ± SEM, *n* = 9, one-way ANOVA with Tukey’s multiple comparison test) significantly increases 4 days following tape strip insult, as does keratinocyte ki67 expression **(C)** (data represent mean ± SEM, *n* = 9, one-way ANOVA with Tukey’s multiple comparison test), suggesting an increase in proliferation is responsible for epidermal thickening. Scale bar: 50 μm. * = *p* < 0.1 and **** = *p* < 0.0001.

Epidermal thickness appeared increased in H&E stained samples (Figure 1B) following tape strip insult compared with 0 tape strip controls. Quantification revealed this is in fact the case, with epidermal thickness increasing significantly with all tape stripped conditions (Figure 2B). Epidermal thickness increased with removal of 2 tape strips, to a greater degree with 5 tape strips and a lesser extent with 10 tape strips, however all conditions were significantly increased compared with the 0 tape strip control.

This relationship between number of tape strips removed and epidermal thickness was mirrored by immunodetection of the proliferation marker ki67. Immunodetection of ki67 increased with number of tape strips (Figure 2A). ki67 is stained green and nuclei are highlighted in blue, images depict the epidermis only. A small number of positive keratinocytes can be observed per field of view in 0 tape strip controls (Figure 2Aa), however this number increases with 2 tape strips (Figure 2Ab) and is particularly prominent with 5 tape strips (Figure 2Ac), as many cells in the *stratum basale* have stained strongly for ki67 expression. The number of ki67 positive cells also appears increased following 10 tape strips (Figure 2Ad) but to a lesser degree than removal of 5 tape strips. Quantification of ki67 positive cells as a percentage of total nuclei (Figure 2C) supports this observation with increasing expression of ki67 with 2 tape strips, culminating at 5 tape strips and being slightly reduced, albeit still significantly elevated with 10 tape strip removal. Following all tape strip conditions, the number of ki67 positive cells was significantly increased compared with 0 tape strip controls. This supports the previous finding of increased epidermal thickness, suggesting the mechanism behind the epidermal thickening is keratinocyte proliferation, as opposed to other means such as altered keratinocyte morphology.

**Figure 3:**
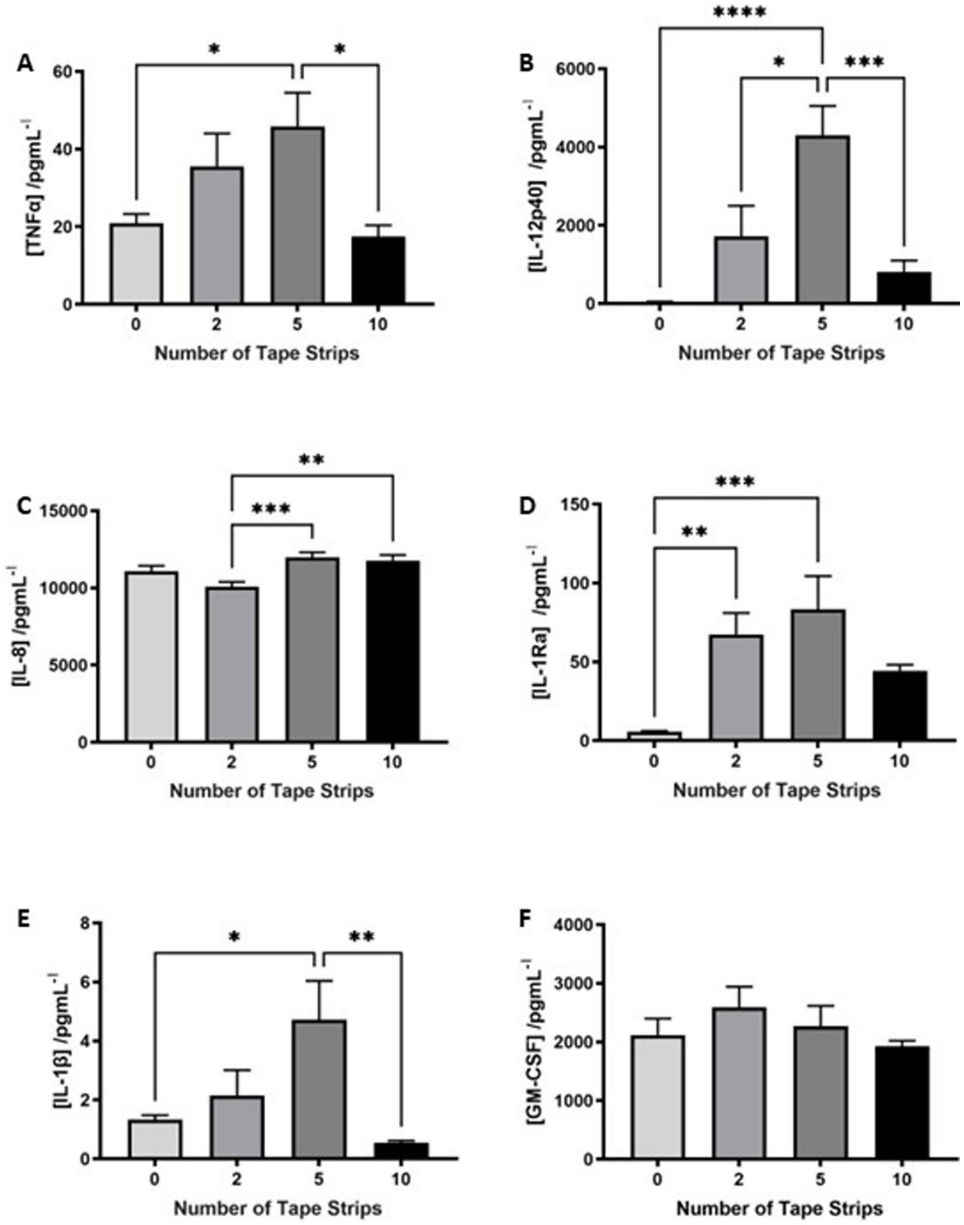
Proinflammatory cytokines are released in response to tape strip insult. An increase in a number of pro-inflammatory cytokines in the culture medium of tape stripped FT-HSEs was detected using a commercially available array. FT-HSEs were tape stripped, returned to culture for 4 days and culture medium harvested for analysis. Pro-inflammatory cytokines including: TNFα **(A)**, IL-12p40 **(B)**, IL-8 **(C)**, IL-1Ra **(D)**, IL-1β **(E)** and GM-CSF **(F)** were all increased in tape stripped samples (data represent mean ± SEM, *n* = 27, one-way ANOVA with Tukey’s multiple comparison test). * = *p* < 0.1, ** = *p* < 0.01, *** = *p* < 0.001, and **** = *p* < 0.0001.

Epidermal thickening is known to be a consequence of inflammatory skin diseases such as atopic dermatitis and psoriasis, therefore, we utilized a commercially available cytokine array to determine the inflammatory state of HSEs following tape strip insult. The concentration of a number of pro-inflammatory cytokines known to be produced by keratinocytes was increased in the culture medium of tape stripped HSEs 4 days following insult, with clear dose-dependent relationships evident in some cases. TNFα (Figure 3A) was increased following 2 tape strips and significantly elevated by 5 tape strip removal, before returning to control levels after 10 tape strip removal. A similar trend was observed with IL-12p40 (Figure 3B) and IL-1Ra (Figure 3D), with medium concentrations increasing slightly with 2 tape strips (significantly in the case of IL-1Ra), to the greatest degree and statistically significantly after 5 tape strips and returning to control levels following 10 tape strip removal. IL-1β (Figure 3E), also followed this trend with concentration increasing slightly following 2 tape strips, significantly after 5 tape strips and returning to control levels after 10 tape strips. GM-CSF (Figure 3F) followed this trend but less markedly with a moderate increase with 2 tape strip removal, decreasing with 5 and 10 tape strip removal. However, IL-8 (Figure 3C) production was enhanced by 5–10 tape strips and slightly reduced by 2. Overall, the increased levels of a number of cytokines in the culture medium following tape strip insult, suggests HSEs are responding to barrier damage through inflammatory mechanisms.

### 3.3. Tape strip removal models aspects of shaving-associated skin damage *in vitro*

**Figure 4:**
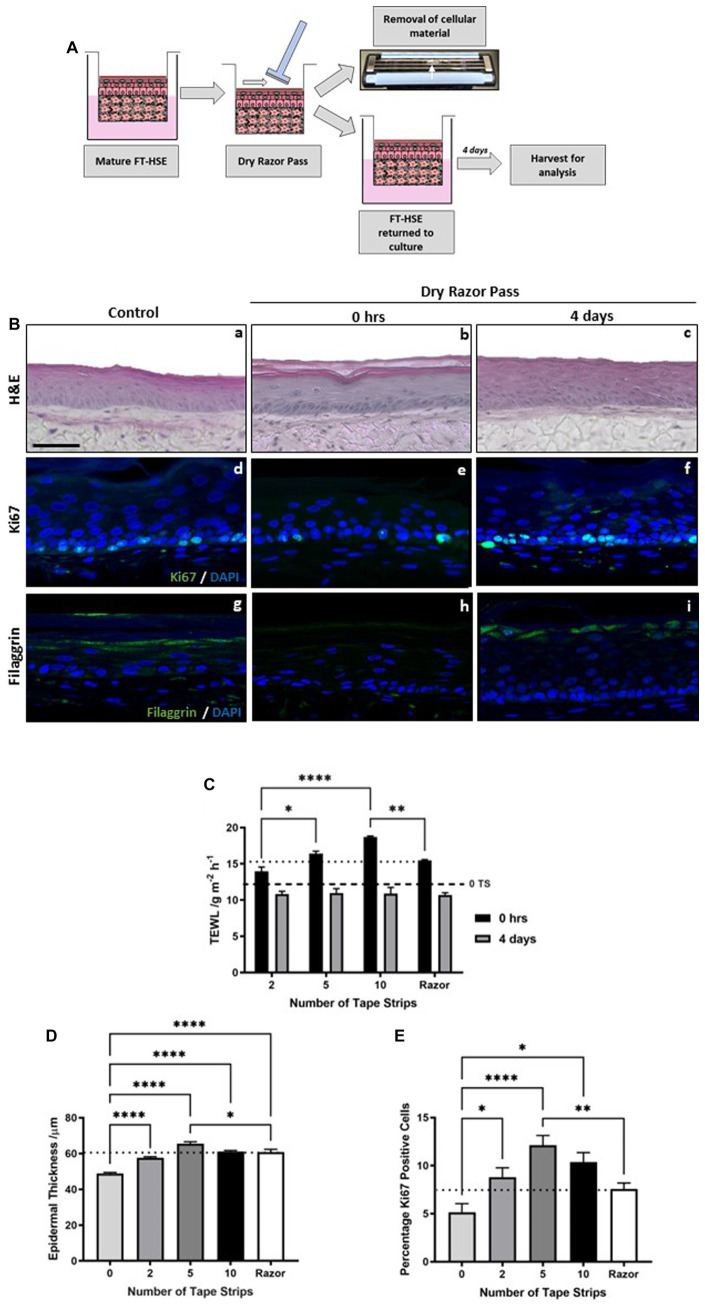
Tape strip induced epidermal barrier damage is comparable to single dry razor pass. Schematic protocol for the dry shaving of FT-HSEs *in vitro* using a disposable razor **(A)**. HSEs were unclipped from culture inserts and a razor was passed over the in the absence of lubrication, cellular material is visible on the blades and models were returned to culture for 4 days prior to analysis. H&E staining **(Ba–c)** of HSEs immediately following a dry razor pass or 4 days post-insult. Immunofluorescence images **(Bd–f)** reveal ki67 positive cells (green) and nuclei (blue), and barrier marker filaggrin (green) with nuclei (blue) **(Bg–i)**. Transepidermal water loss (TEWL) **(C)** (data represent mean ± SEM, *n* = 9, two-way ANOVA with Tukey’s multiple comparison test) increased following razor pass compared with control samples (black dashed line) to a level comparable with severe tape stripping (dotted line), and recovered over a 4 days period. Both epidermal thickness **(D)** (data represent mean ± SEM, *n* = 9, one-way ANOVA with Tukey’s multiple comparison test) and ki67 positivity **(E)** (data represent mean ± SEM, *n* = 12, one-way ANOVA with Tukey’s multiple comparison test) increased following a dry razor pass to a level comparable to mild-moderate tape strip insult (dotted line). Scale bar: 50 μm. * = *p* < 0.1, ** = *p* < 0.01, and **** = *p* < 0.0001.

We hypothesized that exfoliation of the *stratum corneum* through tape strip removal mimics aspects of skin barrier damage induced by razor-based shaving, which not only removes hairs but also has a diffuse impact on the integrity of the skin surface. To test this hypothesis, we passed a standard disposable razor across the surface of HSEs in the absence of lubrication or water (Figure 4A). To enable the razor pass, HSEs were unclipped from their culture inserts, as with tape stripping, and a disposable 3-blade razor was passed across the surface of the HSE in dry conditions. Cellular material was observed on the blades of the razor and HSEs were returned to culture for 4 days before analysis.

Histological analysis reveals that a single dry razor pass did not impact epidermal morphology or viability either immediately following insult or following a 4 days recovery period (Figures 4Bb,c). However, TEWL (Figure 4C) was immediately increased following a dry razor pass to a value that corresponds to approximately 10 tape strips (dotted line). After a further 4 days in culture, TEWL returned to control levels (dashed line), exhibiting a similar recovery timescale as tape strip insult, and again demonstrating the robustness of HSEs.

Immunofluorescence staining for the biomarker filaggrin, reveals a reduction in expression immediately following a single dry razor pass (Figure 4Bh), with restoration of positive staining 4 days post-insult (Figure 4Bi). This evidence supports TEWL measurement data, a functional assessment of barrier function, suggesting structural components that contribute to the skin’s barrier function play a role in the response to mechanical damage.

From the histological images (Figures 4Ba–c) it is noticeable that epidermal thickness appeared greater in the razor pass HSEs than the control HSEs. We confirmed this observation through quantification (Figure 4D), which demonstrated a significant increase in epidermal thickness following a single dry razor pass, a similar trend identified with tape strip removal. The increase in epidermal thickness was similar to all tape strip conditions tested, but almost identical figures were achieved from 10 tape strip removal and a single dry razor pass (dotted line). As with tape strip insult, ki67 positive immunoreactivity appeared enhanced following razor pass (Figures 4Bd–f) and was supported by quantification (Figure 4E). The number of ki67 positive cells was increased following dry razor pass, comparable to that observed from 2 tape strip removal (dotted line).

Together, this data suggests that a single, dry razor pass evokes barrier dysfunction and pro-inflammatory responses, leading to increased keratinocyte proliferation and epidermal thickening, to a similar degree as mild-moderate tape strip removal, validating tape stripping as a method of diffuse barrier damage.

### 3.4. Incorporation of a shaving regimen improves skin health and attenuates inflammation

Skin care products and grooming regimes are often marketed to reduce shaving-induced irritation and restore barrier function following insult, and there is a wealth of evidence to support the role of reduced friction and moisturization in barrier recovery (17, 22, 43–46). We therefore, utilized multiple aspects of a consumer shaving regime, including an in-shave gel and a previously described glycerin-rich basal moisturizing formulation (35, 36) to investigate the importance of these aspects in reduction of insult-related barrier damage.

**Figure 5:**
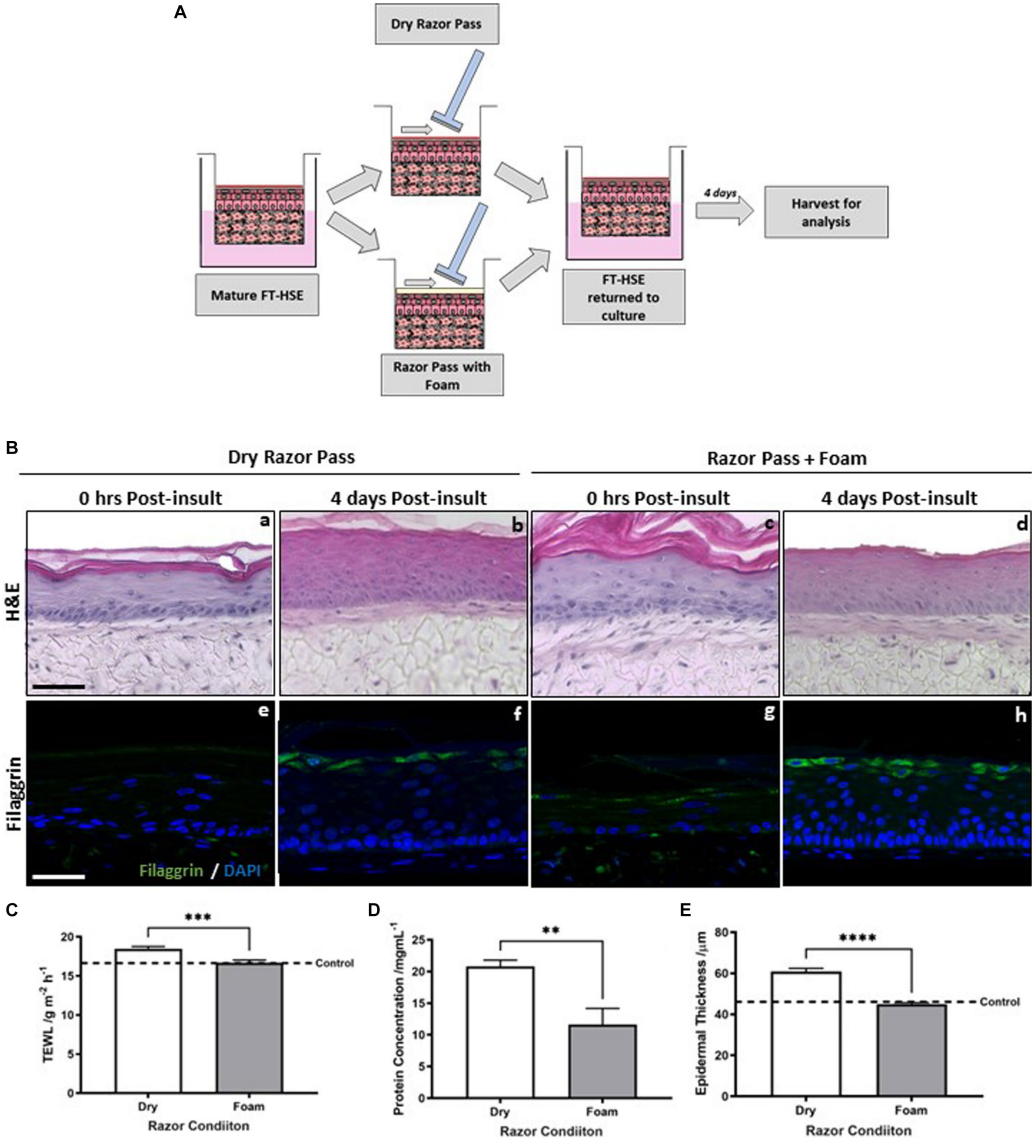
Pre-application of shaving foam reduces razor-mediated skin barrier damage. Schematic protocol for both dry razor shaving and pre-application of shaving foam to FT-HSEs *in vitro* **(A)**. Gillette classic sensitive shaving foam was administered with 10 μL of formulation spread evenly across the surface of HSEs with a glass rod prior to razor-pass. HSEs were either harvested immediately or returned to culture for 4 days post-insult and H&E staining **(Ba–d)** reveals their morphology. Representative immunofluorescence staining of filaggrin, a biomarker of an intact barrier in green and nuclei are highlighted in blue **(Be–h)**. Transepidermal water loss (TEWL) **(C)** (data represent mean ± SEM, *n* = 9, unpaired two-tailed student’s *t*-test) was reduced by pre-application of foam, as was the concentration of protein removed by a single razor pass **(D)** (data represent mean ± SEM, *n* = 9, unpaired two-tailed student’s *t*-test). Epidermal thickness **(E)** (data represent mean ± SEM, *n* = 9, unpaired two-tailed student’s *t*-test) was reduced after a 4 days recovery period when pre-application of shaving foam was incorporated into the regimen. Scale bar: 50 μm. ** = *p* < 0.01, *** = *p* < 0.001, **** = *p* < 0.0001.

Initially, we combined the use of a consumer three blade disposable razor with an in-shave foam to reduce friction and improve lubrication during the shaving process. Mature HSEs were removed from their culture insert (Figure 5A) and either dry shaved or shaving foam applied topically to their surface, evenly spread with a glass rod, before the razor was passed across their surface. HSEs were then returned to culture for 4 days.

Histology reveals that epidermal morphology (Figure 5B) is well organized and viable following both a dry razor pass and with the incorporation of foam into the regime, immediately following razor pass and 4 days post-insult.

Filaggrin immunodetection revealed little staining immediately following a single dry razor pass (Figure 5Be) with more but diffuse and inconsistent staining present immediately following a razor pass with the incorporation of foam (Figure 5Bg). Immunostaining was more visible 4 days post-insult and increased with the presence of foam into the shaving regime (Figure 5Bh).

Immediately post-insult TEWL (Figure 5C) was significantly increased by the dry shave condition, whereas with the addition of shaving foam, TEWL was comparable to the control in the absence of a razor pass (dashed line), suggesting incorporation of shaving foam attenuates razor-mediated barrier damage. Furthermore, the amount of protein (Figure 5D) collected from the blades of the razor and subsequently quantified was significantly greater in the dry shaved condition than with the addition of shaving foam, supporting the increased removal of cellular material and physical damage to the surface of the HSE in the absence of shaving foam. Quantification of epidermal thickness (Figure 5E) revealed a significant decrease in epidermal thickness with the addition of shaving foam in line with the control (dashed line), which is supported by the visible difference observed in the histological staining.

**Figure 6:**
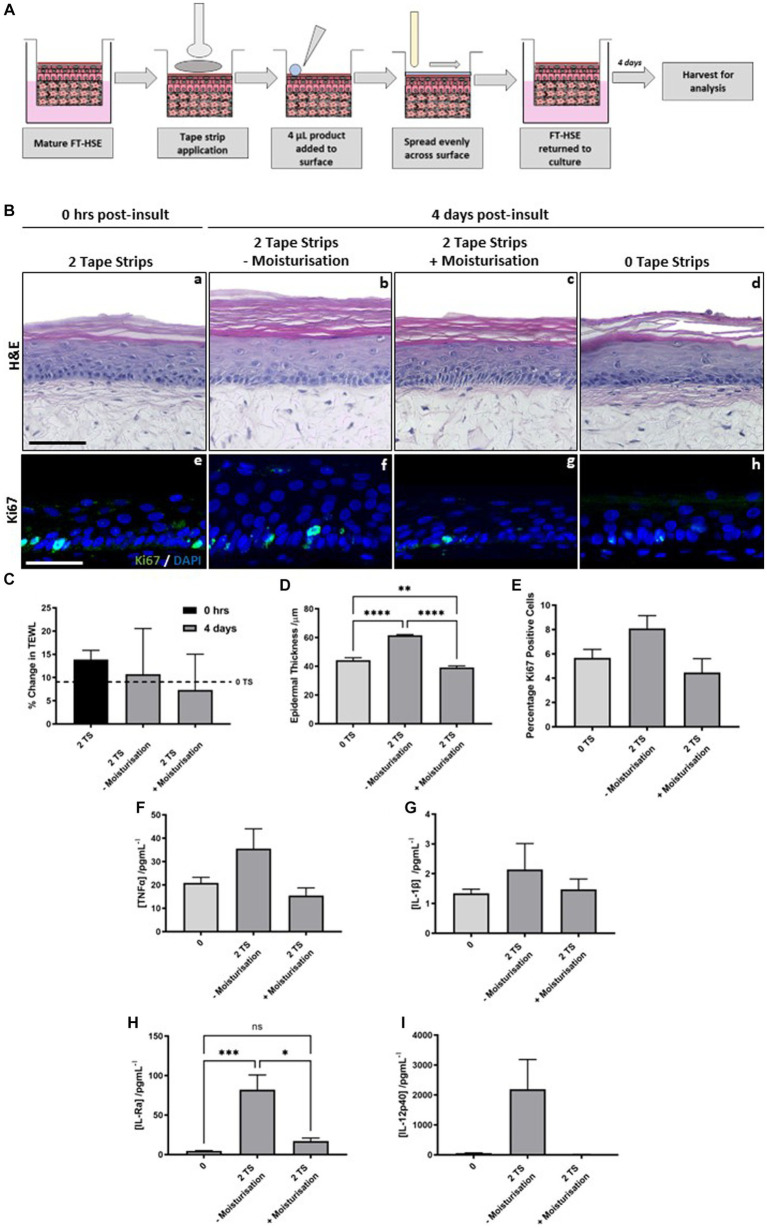
Post-insult moisturisation attenuates tape strip-induced damage. Schematic protocol depicting tape strip application and subsequent topical application **(A)**. Two tape strips were removed from HSEs, a moisturising formulation was added topically to the surface and spread evenly using a glass rod, HSEs were then returned to culture for 4 days. H&E staining of HSEs immediately following removal of two strips **(Ba)** and 4 days post-insult treated with **(Bc)** and without moisturisation **(Bb)** or with no tape strips removed **(Bd)**. Representative images of ki67 staining **(Be–h)** (ki67 green, nuclei blue), demonstrating changes in proliferation marker expression with treatment conditions. Transepidermal water loss (TEWL) **(C)** (data represent mean ± SEM, *n* = 3, one-way ANOVA with Tukey’s multiple comparison test) as a percentage of day 0 measurements. TEWL increases with time in culture without tape stripping (dashed line, 0 TS) but to a greater degree with tape stripping, which is reduced with post-tape strip moisturisation. Epidermal thickness **(D)** (data represent mean ± SEM, *n* = 3, one-way ANOVA with Tukey’s multiple comparison test) and percentage ki67 positive cells **(E)** (data represent mean ± SEM, *n* = 3, one-way ANOVA with Tukey’s multiple comparison test) are both increased with tape strip insult and reduced by post-insult moisturisation. Concentration of pro-inflammatory cytokines: TNFα **(F)**, IL-1β **(G)**, IL-Ra **(H)** and IL-12p40 **(I)** were increased in the culture medium following tape strip insult, and subsequently reduced by moisturisation (data represent mean ± SEM, *n* = 6, one-way ANOVA with Tukey’s multiple comparison test). Scale bar: 50 μm. * = *p* < 0.1, ** = *p* < 0.01, *** = *p* < 0.001, and **** = *p* < 0.0001.

HSEs were removed from culture inserts and 2 tape strips were applied and removed from their surface, then as previously described (35, 36), a small volume of moisturizing formulation was added to the surface of the HSE and spread evenly using a glass rod (Figure 6A). HSEs were then returned to culture inserts and further cultured for 4 days before analysis.

Histology reveals that epidermal structure is unaffected immediately following 2 tape strip removal (Figure 6Ba). The epidermis of HSEs 4 days following 2 tape strip removal appears thicker in the absence of moisturization (Figure 6Bb) compared with those that received topical moisturizing cream application (Figure 6Bc). The epidermal thickness in control HSEs, in the absence of any insult (Figure 6Bd) appears similar to those that received 2 tape strips followed by moisturization. This observation is supported by quantification (Figure 6D), that revealed significant epidermal thickening following 2 tape trip removal, compared with those that did not receive any tape strip insult, which again, was significantly reduced when 2 tape strips were combined with post-insult moisturization. ki67 positivity, mirrored this finding with a greater number of positive keratinocytes visible per field of view following tape strip removal in the absence of moisturization (Figures 6Be–h). Quantification of ki67 positive keratinocytes (Figure 6E) displayed the same trend as that of epidermal thickening, with an increase in ki67 positive keratinocytes following 2 tape strip removal in the absence of moisturization, which was attenuated when HSEs were treated with topical moisturizing cream following insult.

Immediately following tape strip removal barrier function was impaired as determined by an increase in TEWL (Figure 6C) compared with those HSEs that had not been tape stripped (dashed line). After 4 days following insult, TEWL had lowered in HSEs that received 2 tape strips and no moisturization, whereas, TEWL decreased to a greater degree in those HSEs that received both 2 tape strip insult and moisturization. This suggests that post-insult moisturization helps to reduce barrier damage induced by tape strip removal.

Pro-inflammatory cytokine release is increased following tape strip insult, however post-insult moisturization acts to reduce this increase in a number of cases. TNFα (Figure 6F), IL-1β (Figure 6G), IL-Ra (Figure 6H) and IL-12p40 (Figure 6I) all demonstrate the same trend in that they are increased (statistically significantly in the case of IL-Ra) following removal of 2 tape strips in the absence of moisturization and reduced by the combination of 2 tape strip removal with post-insult moisturization. This suggests that post-insult moisturization is acting to dampen down the inflammatory response evoked by tape strip removal, and attenuated some of the negative consequences that arise due to barrier damage.

## 4. Discussion

In this study we have provided evidence for a novel *in-vitro* application of tape stripping to recapitulate the uplift of corneocytes and perturbation of the skin barrier, with subsequent inflammatory responses, observed after razor shaving. Whilst the main consumer aim of the shave process is the removal of hair, the act of shaving can have detrimental side effects to skin barrier properties which can lead to consumer discomfort and irritation if correct technique and regime are not followed (14). The establishment of a standardized *in vitro* method, utilising HSEs to study the impact to skin of shaving, overcomes many of the limiting factors associated with clinical trials such as cost, human error/variability and the limits of using invasive techniques to follow responses over a time course etc. As described here, *in-vitro* studies allow for the in-depth biological analysis of an array of otherwise invasive techniques which can further the understanding of the diffuse skin barrier responses to mechanical disruptions, and subsequently the analysis of ways which can ameliorate these negative impacts. We assess HSE responses to tape stripping and relate results to those induced by razor shaving.

Tape stripping was utilised as a method of barrier disruption because it provides a very well characterised and reproducibly standardised method of *stratum corneum* removal. Indeed, in a study that quantified the amount of *stratum corneum* removed from various sites in human volunteers, it was found that there was no statistical difference in the amount of material removed by each single tape strip analysed from all body sites tested, thereby confirming the standardisation of removal of material (47).

In this study, the removal of 0, 2, 5 and 10 sequential tape strips were taken from HSE to represent no, mild, moderate and extreme levels of barrier disruption. HSEs were shown to withstand each of the levels of tape stripping with no lasting impact to morphology following 4 days of post-tape strip culture, indicating robustness of the skin mimic and suitability for the study. TEWL analysis immediately post tape stripping indicated a corresponding increase in TEWL value relating to the number of tape strips taken, an indicator of barrier damage consistent with the level of damage being induced. These findings are in direct comparison with those found in an *in vivo* study of human volunteers studying barrier damage in relation to the number of tape strip taken (48). TEWL values returned to control levels following 4 days post tape strip culture in all conditions. These data represent evidence HSE barrier properties are damaged to varying levels corresponding to the number of tape strips taken, and in all cases barrier damage is repaired and barrier recovery induced after the 4 days recovery period.

Here we demonstrated through morphological analysis of HSE 4 days post-tape strip, that a thicker epidermis in tape-stripped models is observed when compared to 0 tape strip controls. The most significant increase being in the 5 tape strip, or moderate damage, condition. A corresponding increase in the proliferation marker ki67 was also witnessed. Again the highest increase was seen in the moderate, 5 tape strip condition. Taken together, these data indicate that the mechanical disruption of the skin barrier led to a recovery process involving the proliferation of keratinocytes and resulting in the epidermal thickening observed, which is a known consequence of mechanical skin damage, including post-shave *in vivo* (14). While the 10 tape strip, or extreme damage condition also showed a significant increase in thickening and an increase in the proliferation of keratinocytes above control levels, this was at a reduced amount when compared to the 5 tape strip, or moderate condition. It could, therefore, be inferred that this extreme level of induced barrier damage in the HSEs has led to a reduced or different type of epidermal barrier response to the level of damage induced.

As epidermal thickening and keratinocyte hyperproliferation are known characteristics of skin inflammation observed *in vivo* post-shave (14), we examined the release of pro-inflammatory cytokine markers into the culture media using a commercially available array. It has been well documented that keratinocytes release cytokines in response to barrier damage, wounding and post-shave (12, 20, 49, 50), a range of such cytokines were investigated within this model system. TNF-α, IL-12p40, IL-1RA, IL-1B and GM-CSF were all elevated in the 2, and 5 tape strip conditions indicating that the HSEs were responding to the tape strip induced barrier damage at mild and moderate levels through inflammatory mechanisms in part via keratinocyte stimulation. Indeed, TNF- α is a key component of the inflammatory cascade in skin (51), IL-12p40 acts as a chemoattractant for macrophages (52), IL-1RA and IL-1B are known to be upregulated during inflammatory skin conditions such as psoriasis (50) and finally GM-CSF is known to activate regenerative epidermal growth and stimulate keratinocyte proliferation (53). However, it is important to note that the most extreme damage condition, 10 tape strips, did not follow this upregulated trend, with no increase in cytokine levels observed in these markers when compared to control samples. This could again be an indication of a differing response of HSEs to the extreme level of damage. It should also be noted that the cytokine analysis presented here only provides a “snapshot” of expression levels at the 4 days post insult time point. Therefore, to carry out a full, in-depth analysis of inflammatory cytokine markers present in the different levels of induced damage, a more comprehensive time course study would need to be conducted, but is outside the scope of this present investigation. IL-8, a member of the supergene family of proinflammatory and chemotactic cytokines, however, was significantly up-regulated in both the 5 and 10 tape strip conditions, an indication that some inflammatory response is occurring in the 10 tape strip, extreme damage condition also.

The next set of experiments aimed to address the hypothesis that the standardised barrier damage induced via the tape strip methodology mimics the barrier damage induced by dry razor shaving, thus validating the model platform for further in-depth shave-related investigations. Indeed, when HSEs were exposed to a single dry razor pass the morphometrics analysed placed the damage induced, and the responses tested in line with the removal of between 2 and 5 tape strips. Therefore, the level of damage induced by one pass with a dry razor with 5 blades induced mild to moderate barrier damage followed by pro-inflammatory responses, leading to increased keratinocyte proliferation and epidermal thickening. These data further support the use of the platform technology for shave-related investigations. Furthermore, following analysis of 4 days post razor conditions full barrier recovery was observed, as expected.

Finally, to investigate the use of the novel platform technology for shave-care regime investigations and screening, the pre-application of shaving foam, and the post-tape strip application of a moisturising cream was studied.

Pre-shave application of a shaving foam to the model surface, when directly compared with dry shave conditions was found to have a protective effect to the barrier, attenuating some of the damage measured with the dry shave. This result is as expected, and is proof of concept that the models respond to grooming applications in the same manner as *in vivo* human skin.

The topical application of the moisturiser reduced the level of epidermal thickening and keratinocyte hyperproliferation down to near control levels when applied immediately following a mild level of tape stripping. This level of mild tape stripping was selected as when tape stripping outcomes were compared with a single dry razor pass, removal of 2 tape strips correlated well with razor damage for those end points measured. TEWL values observed 4 days post tape strip were reduced in the models that received moisturisation. These findings indicated the enhanced barrier recovery induced by moisture application to damaged skin, a well-documented phenomenon (17, 22, 43–46), and provides proof of concept for the platform technology herein. Indeed, the levels of the pro-inflammatory cytokines that were shown to be enhanced following tape stripping were significantly reduced in post tape strip moisturisation application models. These findings indicate that the application of a moisturiser post-shave dampens the inflammatory responses and leads to an attenuation of the negative impacts to the skin barrier following a mimic of corneocyte uplift.

Furthermore, we demonstrate the robustness of our model HSE system to sustain extreme levels of barrier damage, remain intact and ultimately recover to some extent. We also outline tape stripping as a proposed *in vitro* method that recapitulates aspects of the shaving response including: inflammation, hyperproliferation, barrier disruption and epidermal thickening. As a model system, this allows the quantification of such end points to screen a wide range of shaving regimens to determine their effect on skin health. With particular interest, we propose epidermal thickness as an end point measure, which correlates well with pro-inflammatory markers, has provided statistically significant quantifiable data and can be automated for high throughput screening. This methodology also allows flexibility in that the number of tape strips removed correlates with the level of damage observed, and can represent barrier damage acquired from a range of shaving regimens.

Although the current study demonstrates the robustness of our HSE system to investigate diffuse barrier damage and recovery, there are however some limitations to the approach. Namely, the HSE described herein comprises of keratinocytes, fibroblasts and endogenous ECM, whereas native skin also contains appendages and supporting cell types. Inclusion of cell types such as immune cells that mediate inflammatory responses, melanocytes to model post-inflammatory hyperpigmentation (34) or a neurosensory component to represent irritation (35), could provide further insights into the complicated damage response of skin as a complex organ. Furthermore, the absence of a hair follicle in our HSE system, limits its use for studying shaving regimes, however in this study we describe novel approaches that utilise both tape stripping and razor-based shaving, focussing on the specific effects on skin barrier function and surface damage, rather than hair removal efficacy.

Despite its limitations, we present compelling proof-of-concept data that tape strip removal of material from the *stratum corneum* can represent barrier damage comparable to that induced by razor-based shaving. We also present the novel use of HSE-technology to assess razor-induced skin damage *in vitro*, therefore the methodologies we have described, provide a platform for future studies. We demonstrate the beneficial action of both in-shave foam and post-shave moisturisation to negate negative effects on the skin barrier. Combining these technologies, we have presented a novel system that lends itself to a broad range of future studies including pre-clinical screening of commercial razor types and whole regimes to determine their beneficial effects on skin barrier function.

This study has demonstrated the use of tape strip techniques on a well characterised and defined HSE to mimic the barrier disruptions induced by dry razor shaving. Data shown herein demonstrate proof of concept for the use of the model platform technology to further study the protective and restorative effects of elements of a shave care regimen either individually or as a full shave care investigation.

## Data availability statement

The raw data supporting the conclusions of this article will be made available by the authors, without undue reservation.

## Ethics statement

Ethical approval was not required for the studies on animals in accordance with the local legislation and institutional requirements because only commercially available established cell lines were used.

## Author contributions

LC, KG, VM, IA, TD, EH, AS, and SP contributed to conception and design of the study. LC, KG, VM, and NB were largely responsible for the experimental plan and experimental work. LC, KG, and VM wrote the first draft of the manuscript with input from NB, KS, IA, EH, TD, AS, and SP. All authors contributed to the article and approved the submitted version.

## Footnotes

^1^http://imagej.nih.gov
